# Quantifying interflake ordering in graphene oxide films via wide-angle X-ray scattering analysis

**DOI:** 10.1107/S1600576725008714

**Published:** 2025-11-26

**Authors:** Roque Sanchez Salas, Aaron Morelos Gomez, David Angel Sanchez Hernandez, Sandra Loera Serna

**Affiliations:** a Departamento de Ciencias Básicas Universidad Autónoma Metropolitana Unidad Azcapotzalco Av San Pablo 420 Col Nueva el Rosario Azcapotzalco CP 02128 Ciudad de México Mexico; b ElectNano, 3850 E Baseline Rd Ste 125, Mesa, AZ85206, USA; c Departamento de Metal Mecánica, Instituto Tecnológico de San Luis Potosí, Av. Tecnológico S/N, Col. Unidad Ponciano Arriaga, Soledad de Graciano Sánchez, San Luis Potosí 78437, Mexico; Brazilian Synchrotron Light Laboratory, Brazil

**Keywords:** wide-angle X-ray scattering, graphene oxide films, anisotropy quantification

## Abstract

We report a methodology based on wide-angle X-ray scattering (WAXS) to quantify the ordering between graphene oxide (GO) flakes in self-assembled films. By comparing WAXS signals of GO and graphite, we extract orientation distribution coefficients (*S*) from the GO 001 and (100) scattering features. To characterize the anisotropy in flake alignment, we define a ratio parameter (*R*) as the quotient of orientation distribution coefficients obtained under orthogonal orientations from the normal direction of the GO film with respect to the X-ray beam. This dimensionless ratio allows us to assess the degree of meso­structural anisotropy and condenses comparative orientation information into a single dimensionless metric, providing a new structural parameter for GO and related layered materials. In our measurements, *R* reached values of 0.11 for the GO 001 signal. These results demonstrate a metric for characterizing anisotropy in disordered 2D materials.

## Abstract 2

We present a methodology based on wide-angle X-ray scattering for the quantitative assessment of interflake ordering in graphene oxide films, providing a structural parameter to characterize anisotropy in disordered 2D materials.

## 1. Introduction

Graphene oxide (GO) is a chemically modified 2D material obtained through oxidative treatment of graphite. Its structure incorporates oxygen-containing functional groups, including hydroxyl, ep­oxy, carbonyl and carboxyl, located on the basal planes and edges of individual flakes. These functionalities significantly affect GO’s chemical behavior, its dispersibility in aqueous media and interflake interactions, enabling a wide range of applications from membranes to composites. Structurally, the inclusion of these groups results in a disordered carbon lattice with randomly distributed vacancies and defects, leading to non-uniformity in both chemical composition and spatial organization. Hydroxyl and ep­oxy groups are typically located on the basal plane, altering reactivity, while carbonyl and carboxyl groups dominate the edges or internal defect sites. The resulting material exhibits local distortions in its *sp*^2^ carbon framework and mesoscopic porosity.

GO lacks a defined crystallographic unit cell for several fundamental reasons: (i) compositional irregularity in elemental content (C, H, O); (ii) the absence of periodic long-range ordering within single flakes, primarily due to oxidative fragmentation; and (iii) disruption of the *ABAB* interlayer stacking typical of graphite, owing to steric effects introduced by the oxygen functional groups. Given these deviations from crystallinity, various descriptors have been proposed to describe GO’s structure. Some authors refer to GO as a quasi-two-dimensional crystal, while others liken it to a liquid crystal when considering the collective orientation of flakes in dispersions (Jalili *et al.*, 2014 ▸; Wu *et al.*, 2024 ▸). However, these terms remain conceptual, as GO lacks strict periodicity and long-range order in the solid state.

In the literature, powder X-ray diffraction (XRD) in the Bragg–Brentano configuration utilizing point detectors is frequently employed to verify GO synthesis. Typically, the disappearance of graphite (002) peaks and the appearance of a broad signal commonly attributed to the ‘GO characteristic peak (001)’ are used as indicators of a successful graphitic precursor oxidation.^1^ However, this interpretation is problem­atic. The GO 001 signal originates from the average interflake spacing, which ranges from 9 to 11 Å and is influenced by factors such as hydration level, oxygen content and flake compaction (Cho *et al.*, 2017 ▸; Devanathan *et al.*, 2016 ▸). This signal exhibits no true crystallographic indexing, as its shape and position vary significantly depending on local mesostructural conditions. In particular, the FWHM of the GO 001 signal is sensitive to flake alignment and the self-assembly process during film deposition. Values of the FWHM can range from 1.8° to 0.5° for the same GO dispersion, depending on conditions that promote flake reorientation (Zhong *et al.*, 2018 ▸). Moreover, experimental studies have shown that the insertion of molecules or ions between GO flakes alters the interflake spacing (Iakunkov *et al.*, 2019 ▸; Liu *et al.*, 2018 ▸; Natkaniec *et al.*, 2015 ▸). This confirms that GO does not meet the requirements for a crystalline material and that conventional XRD interpretations are insufficient to describe its structural behavior.

To overcome these limitations, wide-angle X-ray scattering (WAXS) provides a more suitable tool for structural analysis of disordered 2D materials such as GO (Saurel *et al.*, 2019 ▸; Li *et al.*, 2020 ▸). Unlike conventional XRD instruments in Bragg–Brentano geometry equipped with point detectors, WAXS experiments combined with a 2D area detector enable azimuthal integration of the scattered intensity. In addition, the WAXS sample holder permits controlled placement of flakes and films with a defined orientation relative to the incident beam, which is essential for distinguishing parallel and perpendicular configurations, allowing the assessment of orientational order within flake assemblies. This is particularly relevant for GO films formed by solution casting after drying, where mesostructural anisotropy could emerge.

Common deposition techniques such as drop casting, bar coating and vacuum filtration typically rely on self-assembly during drying, but achieving precise flake alignment with reproducible mesostructures at the macroscopic scale remains challenging. Xin *et al.* (2019 ▸) demonstrated that using flat microfluidic channels enables controlled alignment of GO sheets into belts, tubes and rods through shape confinement, leading to improved electrical, thermal and mechanical performance after reduction. Likewise, Poulin *et al.* (2016 ▸) showed that shear flow induces pronounced alignment of GO sheets, transforming them from crumpled to flattened states, with a notable decrease in the layer spacing. The exceptionally low bending rigidity of GO (∼1*kT* ), which is about two times lower in thermal energy than the bending rigidity of neat graphene, facilitates structural rearrangement under shear, enabling aligned structures with enhanced mechanical strength, thermal stability and electrical conductivity (Zhong *et al.*, 2018 ▸), as well as selective permeability in filtration processes (Akbari *et al.*, 2016 ▸).

Small-angle X-ray scattering (SAXS) has been used to probe orientational order in GO dispersions, films and fibers (Wu *et al.*, 2014 ▸; Lu *et al.*, 2017 ▸; Wychowaniec *et al.*, 2021 ▸; Xin *et al.*, 2019 ▸; Poulin *et al.*, 2016 ▸; Shim & Kim, 2023 ▸; Xu & Gao, 2011 ▸), while WAXS has proved to be particularly suitable for mesostructured films (Li *et al.*, 2020 ▸). In both cases, 2D scattering patterns reveal changes in azimuthal intensity distributions, where enhanced or diminished signals along perpendicular directions indicate preferential alignment of GO sheets with respect to the incident beam. The orientational distribution coefficient *S*, derived from azimuthal integration of the scattered intensity, provides a quantitative measure of the degree of alignment, with higher anisotropy reflected in larger *S* values.

In this work, we propose a WAXS-based methodology to characterize the orientational ordering between GO flakes in self-assembled films. The novelty of this study lies in establishing a WAXS-based methodology that combines calibration with crystalline graphite and the definition of a dimensionless anisotropy ratio *R* [equation (3)]. By contrasting orthogonal sample orientations, this approach overcomes the ambiguity of absolute order parameters and provides a robust metric for mesostructural alignment in GO films from the WAXS GO 001 signal (Saurel *et al.*, 2019 ▸; Pfaff *et al.*, 2019 ▸; Burian *et al.*, 1999 ▸), thus enabling us to quantify flake alignment as a function of sample orientation relative to the X-ray beam. This approach enables direct assessment of mesostructural organization in GO and similar non-crystalline layered systems. However, in light of the analysis of the orientation parameter *S* for the (100) reflection, we decided not to estimate an *R* value from this signal. The reason is that the intensity of the (100) reflection in GO predominantly arises from residual *sp*^2^ nanodomains that persist within the flakes, rather than from modifications in the anisotropy of the basal planes. Consequently, the (100) signal is not a reliable descriptor of mesostructural alignment in the films and we instead focus on reflections more directly linked to the basal-plane stacking to quantify anisotropy.

## 2. Experimental materials and methods

GO was synthesized using a modified Hummers method, employing natural graphite flakes (Asbury Carbons, >99% purity, mesh No. 32) as the graphitic precursor, following the protocol described by Sánchez-Salas *et al.* (2021 ▸). After the synthesis, GO was used to prepare the films without any further exfoliation step. In a typical film deposition, a 1.8 mg ml^−1^ aqueous GO dispersion was drop-cast onto a flat polyvinyl­idene fluoride (PVDF) mat until the cast dispersion diameter was approximately 15 cm, then dried overnight on a hot plate at 70°C. The resulting films could be easily peeled off the substrate. The elemental carbon-to-oxygen (C/O) atomic ratio of the pristine GO films was 2.1. The areal mass density was 1.6 mg cm^−2^ and the average film thickness was 7 µm.

WAXS graphite measurements were performed employing a Kapton tape from both sides of the central hole of the sample holder to fix flakes in random, parallel and perpendicular orientations with respect to the incident X-ray beam. Scattering patterns were background corrected by subtracting the signal obtained from the Kapton tape blank measurement. We also processed the scattering pattern for a subtraction scale factor of 0.96 after background subtraction, since the single graphite flake signal was weak.

WAXS GO measurements were carried out in air and then background corrected by subtracting the signal obtained from an air blank measurement. The GO films were folded into eight layers from a GO strip film of approximately 5 × 10 mm in dimension. For perpendicular orientations of the normal direction of the film with respect to the incident X-ray beam, they were then fixed into the central hole of the sample holder using tweezers and applying a very small pressure. For parallel orientations, they were stacked with tape outside the central aperture (Fig. S1 in the supporting information).

Patterns were collected on beamline BLS53 of the Aichi Synchrotron Radiation Center in transmission geometry using a monochromatic X-ray beam with a wavelength of 0.92 Å under ambient laboratory conditions. The sample-to-detector distance was 201.847 mm. Instrument calibration was performed by the beamline staff prior to the measurements. The samples were mounted on a WAXS sample holder with a 3 mm diameter circular aperture and measurements were acquired through the exposed area. Data processing and azimuthal integration were performed using the *FIT2D* software package, developed by the European Synchrotron Radiation Facility (ESRF) (Hammersley, 2016 ▸); see the video in the supporting information for a guide to the technical details of how azimuthal integration is done for a graphite flake in the [100] zone axis parallel to the beam.

The degree of alignment was quantitatively estimated using an orientation distribution coefficient *S*, which was defined by Hermans’ orientation function as the mean of the second order of the Legendre polynomial:
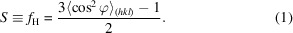
Then for a set of (*hkl*) planes, this orientation distribution coefficient can be correlated with the azimuthal dependence of the scattered intensity,
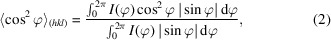
where φ is the azimuthal angle, *I*(φ) is the scattered intensity along the angle φ and the azimuthal integration is over 2π with ∣sin φ∣ weighting (equivalent to folding to 0–180° with sin φ).

*S* has a value of unity when the normal of the reflection plane is parallel to the beam reference direction, a value of zero when there is random orientation in the sample and a value of −0.5 when the normal of the reflection plane is perpendicular to the beam reference direction. The values of the weighted average over the intensity distribution along the diffraction ring 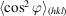 are unity when the normal of the plane is oriented parallel to the beam reference direction and zero when the normal of this plane is perpendicular to the beam reference direction (Ran *et al.*, 2001 ▸).

## 3. Results

To give a structural comparison of the orientational ordering of flakes in graphite and GO, it is useful to move from classical Bragg–Brentano X-ray diffraction using point detectors to WAXS and from loose powders to stacked flakes in freestanding films (Saurel *et al.*, 2019 ▸). To quantify the degree of flake alignment, WAXS is particularly appropriate given the typical interlayer distances of 9–11 Å in GO (Pfaff *et al.*, 2019 ▸).

**Figure 1:**
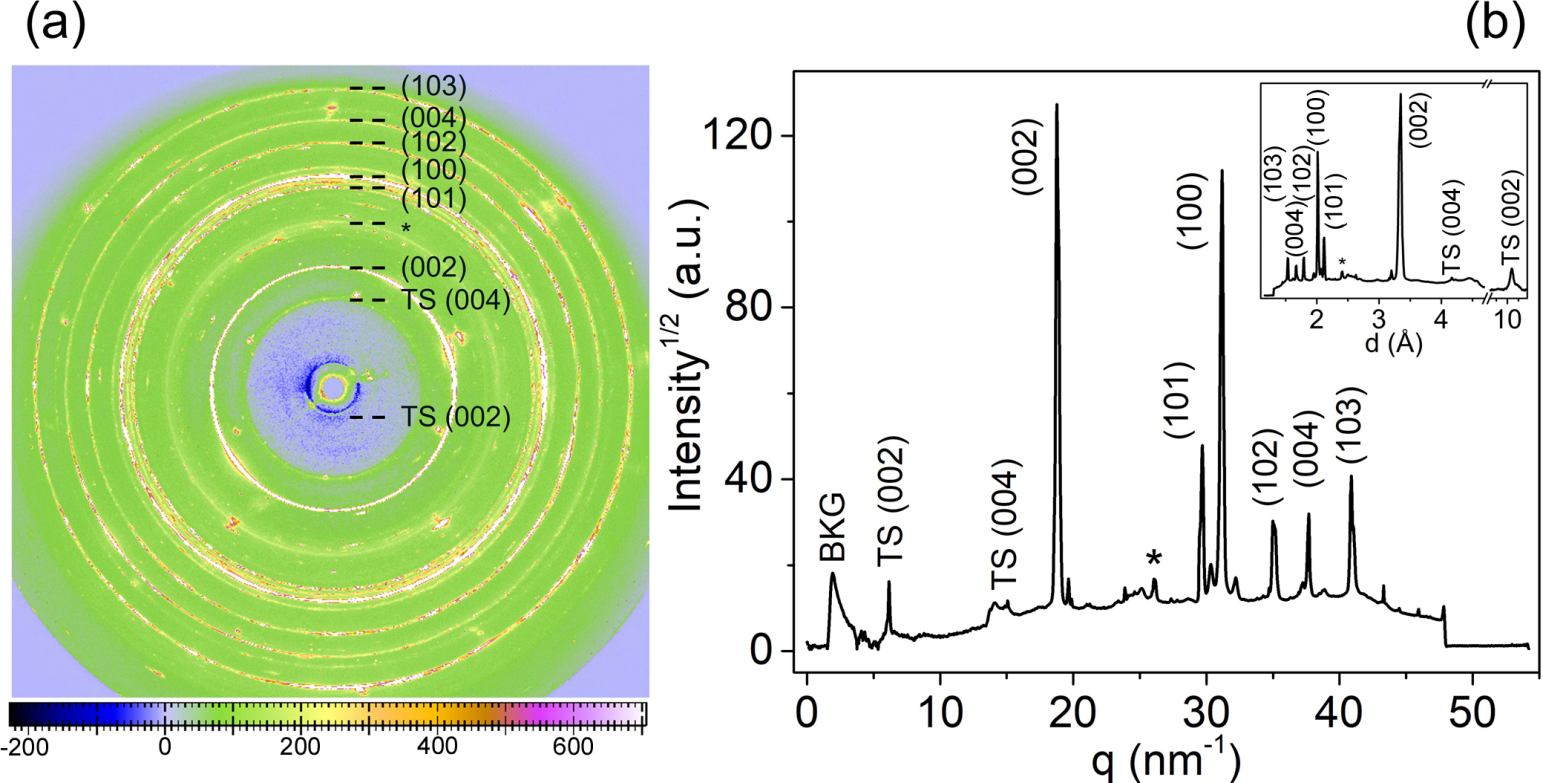
(*a*) WAXS pattern of randomly oriented graphite flakes showing concentric diffraction rings indexed to their corresponding crystallographic planes. (*b*) Azimuthally integrated diffractogram plotted as scattering intensity versus scattering vector magnitude *q*. The inset shows the same diffractogram plotted as a function of interplanar spacing *d*. The scattering features and the integrated diffraction pattern were indexed according to the corresponding crystallographic planes of graphite.

To establish a methodology for analyzing flake ordering in a 2D material, we first examined WAXS patterns from a single mineral graphite flake, which serves as a crystalline refence for the *ABAB* stacked structure. Fig. 1 ▸(*a*) shows the WAXS pattern from a graphite flake placed randomly on the sample holder, which mimics a powder-like ring distribution. Radial integration using *FIT2D* (Hammersley, 2016 ▸) produced a diffractogram as a function of the scattering vector magnitude *q* and interplanar distance *d*, as shown in Fig. 1 ▸(*b*). The main reflections were indexed according to graphite reference data.

**Figure 2:**
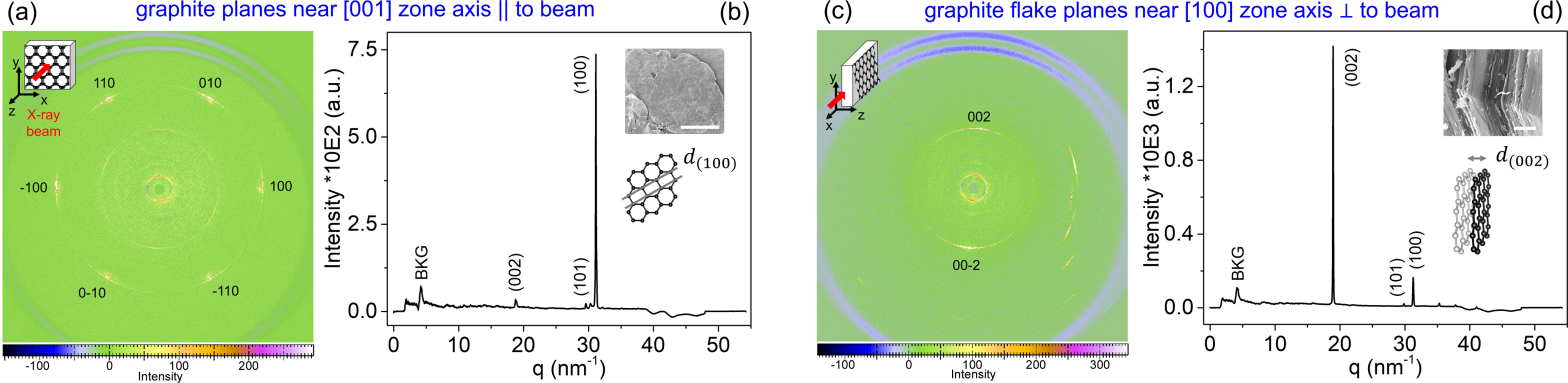
WAXS patterns for a single graphite flake aligned in (*a*) a face-on configuration, such that its basal planes were nearly perpendicular to the incident X-ray beam, corresponding to the [001] zone axis parallel to the beam, and in (*c*) an edge-on configuration, such that its basal planes were nearly parallel to the beam, corresponding to the [100] zone axis perpendicular to the beam. The corresponding scattering features are indexed according to the graphite crystal family reflections. Panels (*b*) and (*d*) display the azimuthally integrated diffraction profiles from the regions containing the indexed scattering features, plotted against the scattering vector magnitude *q*. The inset scanning electron micrographs show face and edge perspective views of the graphite flake for better clarification of the face-on and edge-on settings; their scale bars represent 500 and 1 µm, respectively. The red arrows indicate the direction of the incident X-ray beam.

A highly oriented graphite flake (mesh No. 32) was manually oriented with the [001] zone axis nearly parallel to the beam (*i.e.* basal planes are perpendicular to the beam), which is also called the face-on setting due to the face view of the graphite flake [Fig. 2 ▸(*b*)]. This configuration shows a scattering pattern of the {100} family and the expected sixfold symmetry [Fig. 2 ▸(*a*)], consistent with the hexagonal in-plane reflections (100), (010), (110), (100), (010) and (110) which are equivalent in-plane orientations, and also with a minor contribution from the (002) plane, probably due to edge defects or slight mis­alignment.

A second graphite flake was manually oriented with the [100] zone axis nearly perpendicular to the beam (*i.e.* basal planes are parallel to the beam), which is also called the edge-on setting due to the edge view of the graphite flake [Fig. 2 ▸(*d*)]. The resulting WAXS pattern [Fig. 2 ▸(*c*)] exhibits a two-spot feature with 180°/φ azimuthal symmetry attributed to the (002) and (002) reflections from the basal-plane stacking.

**Figure 3:**
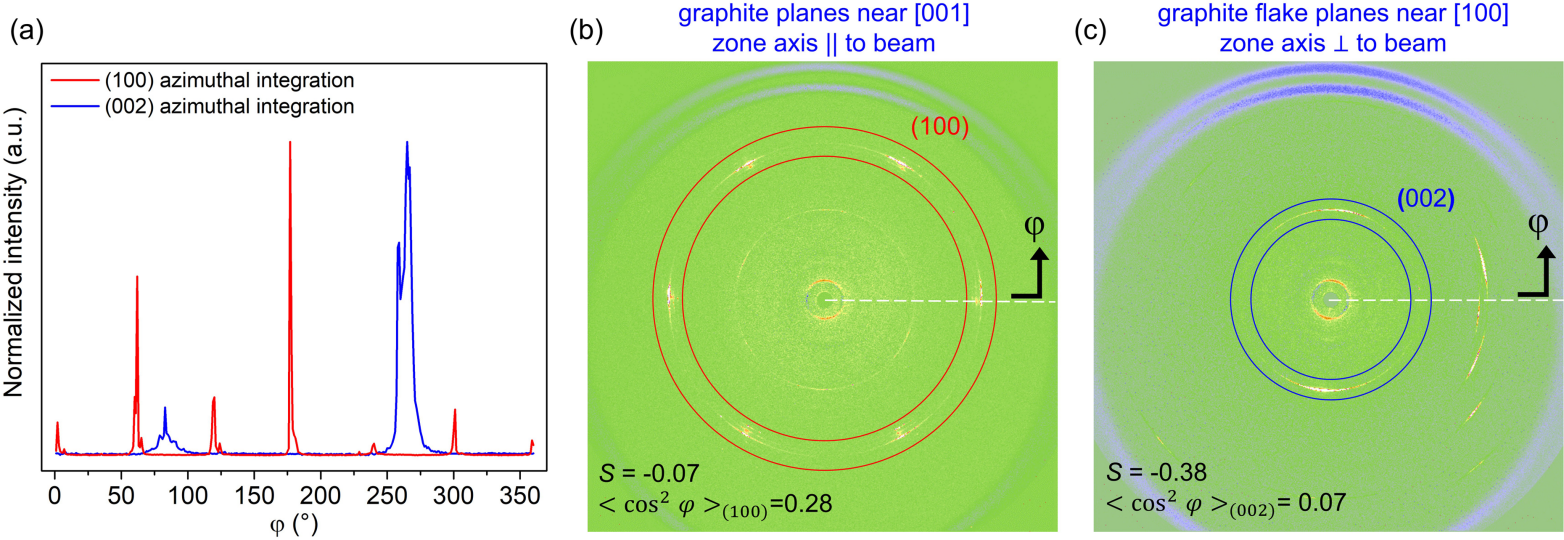
(*a*) Normalized intensity from the X-ray scattering signal as a function of the azimuthal angle φ, obtained by azimuthal integration of the scattered intensity *I*(φ). The data correspond to scattering patterns of a graphite flake oriented (*b*) near the [001] zone axis, with its basal planes perpendicular to the incident X-ray beam, and (*c*) near the [100] zone axis, with its basal planes parallel to the beam. At the bottom, the corresponding values of *S* and 〈cos^2^φ〉_(*hkl*)_ are listed for the cases where a crystallographic plane is oriented with respect to the incident beam.

Fig. 3 ▸(*a*) shows the azimuthal integration from graphite flake aligned settings (face-on and edge-on) with respect to the beam. The video in the supporting information records the procedure to obtain output data from the edge-on setting using *FIT2D*, while the text file in the supporting information demonstrates how *S* and 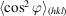 were computed via equations (1) and (2), respectively. In the [001] geometry, where the *c* axis of graphite is parallel to the incident beam and the basal planes are perpendicular to it, the WAXS pattern of the {100} family exhibits the expected sixfold symmetry associated with the hexagonal in-plane lattice. However, when the entire {100} Debye ring is integrated azimuthally [Fig. 3 ▸(*b*)], the averaging over all six equivalent spots yields 〈cos^2^ φ〉_(100)_ = 0.28, corresponding to *S* = −0.07. This apparent ‘near-random’ value does not indicate a lack of crystallographic order but rather reflects the intrinsic rotational degree of freedom within the basal reflections: (100), (010), (110), (100), (010) and (110), which are equivalent in-plane orientations along the *a* and *b* graphite crystallographic directions, remain symmetrically equivalent. As a result, parameters such as *S* approach values close to zero for reflections with multiple equivalent in-plane orientations.

We also examined the [100] geometry [Fig. 3 ▸(*c*)], where the beam is parallel to the *a* axis and the *c* axis is perpendicular to it. In this configuration, the (002) reflection dominates the pattern, appearing as a characteristic meridional two-spot distribution. Azimuthal integration of this reflection yielded 〈cos^2^ φ〉_(002)_ = 0.07, corresponding to *S* = −0.38, which is approaching the theoretical limit of −0.5 expected for a perfectly perpendicular alignment. This result demonstrates that, when the geometry constrains a unique orientation axis, the *S* parameter behaves consistently with theory and provides a reliable measure of anisotropy. Together, the [001] and [100] cases illustrate both the limitations of *S* in the presence of multiple equivalent in-plane orientations and its robustness when applied to reflections with a single preferential axis.

**Figure 4:**
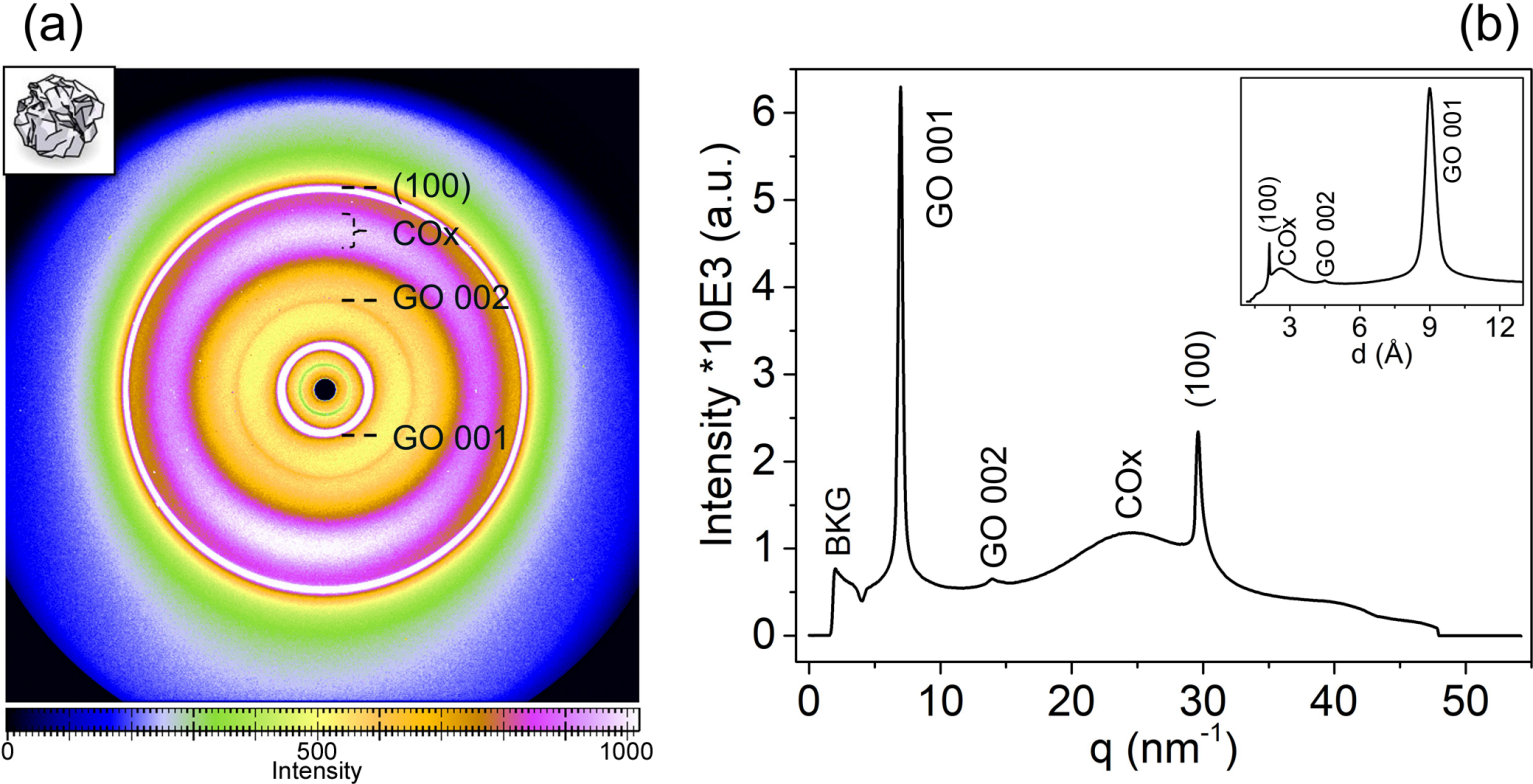
WAXS pattern of a GO paper ball exhibiting random in-plane and out-of-plane flake orientations. (*a*) Scattering features appear as continuous rings, indicating a lack of preferential alignment within the sample. (*b*) The corresponding integrated diffraction pattern as a function of the scattering vector magnitude *q*. The inset in (*b*) shows the same azimuthally integrated pattern plotted against the interlayer spacing *d* in ångströms. No crystallographic indexing is applied, except for the in-plane (100) graphite reflection, which is commonly observed in disordered graphene oxide systems.

Using this calibration based on graphite monocrystals, we then examined randomly folded GO paper as a crumpled ball of GO film to analyze the azimuthal scattering distribution. This WAXS pattern from randomly folded GO paper [Fig. 4 ▸(*a*)] showed multiple rings. After radial integration with key signals labeled for clarity [Fig. 4 ▸(*b*)], the most intense ring (GO 001) corresponds to an interflake spacing of 9.0 Å. A second-order reflection (GO 002) appears at 4.5 Å, while a broader ring around 2.6 Å, labeled COx, is attributed to *sp*^3^-hybridized carbon atoms bound to oxygen functional groups, consistent with the literature (Schniepp *et al.*, 2006 ▸). This COx signal shows 180°/φ azimuthal symmetry, consistent with the bilaterally decorated structure of GO flakes (Natkaniec *et al.*, 2015 ▸). Finally, a ring at 2.1 Å is observed, corresponding to the in-plane (100) graphite reflection, suggesting the persistence of nanodomains with *sp*^2^ hybridization within the GO flakes. Notably, the COx signal is typically absent in conventional Bragg–Brentano geometry XRD with point detectors, due to structural distortion and misalignment, but it is clearly revealed here via WAXS.

## 4. Discussion

Up to this point, we have described the interpretation of the WAXS pattern of GO and the analytical methodology applied to a 2D crystalline material. We now address the question, how can X-ray scattering be used to quantify the orientational ordering among GO flakes?

**Figure 5:**
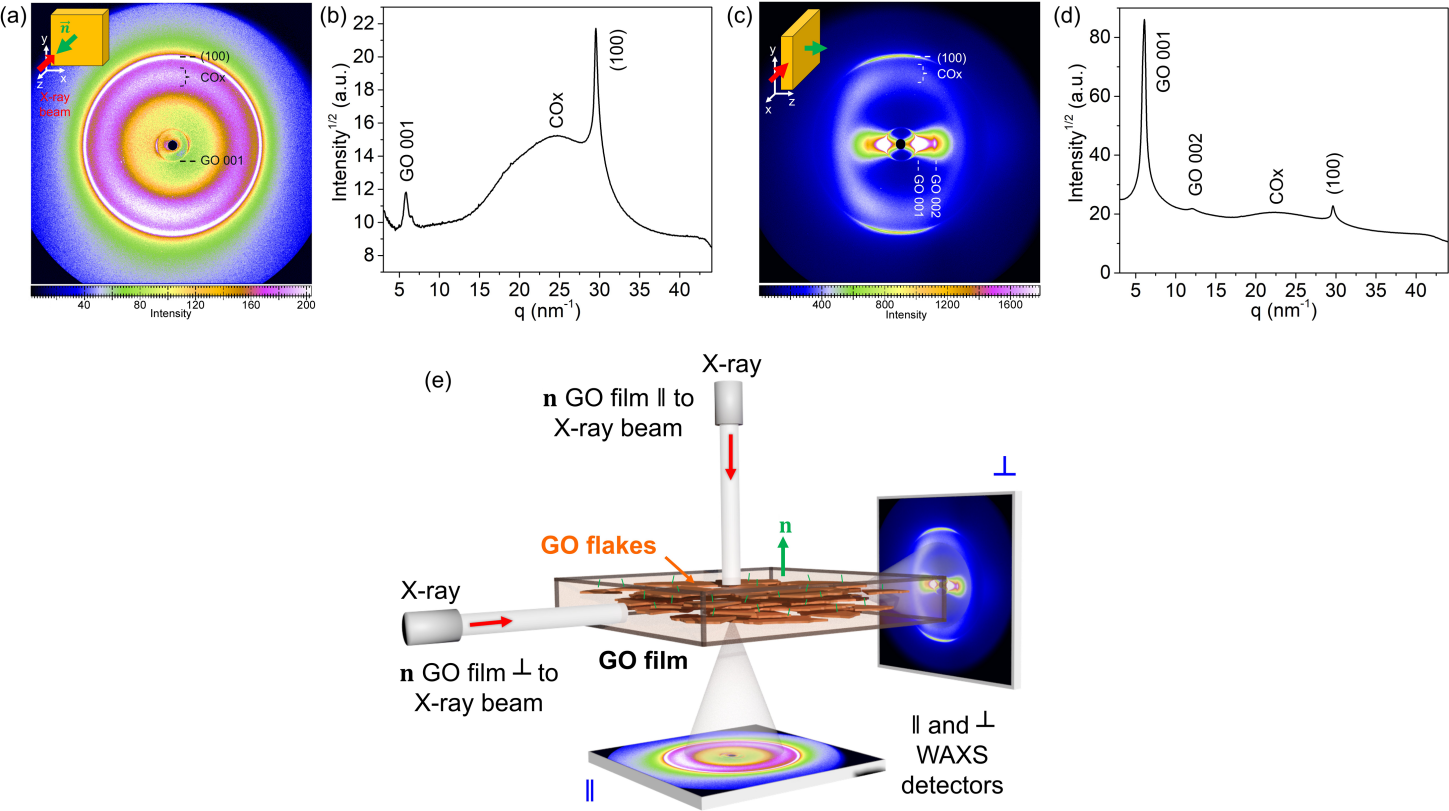
WAXS patterns of a GO film oriented with its normal vector **n** (*a*) parallel to the incident X-ray beam and (*c*) perpendicular to the beam. (*b*) and (*d*) The corresponding azimuthally integrated diffraction patterns plotted as a function of the scattering vector magnitude *q*. The main scattering features associated with GO are labeled in both the 2D WAXS images and the 1D integrated patterns. (*e*) Schematic representation of the GO film orientation relative to the incident X-ray beam, showing configurations where the film’s normal vector **n** is either parallel or perpendicular to the beam and the corresponding contrast observed in their WAXS patterns.

**Figure 6:**
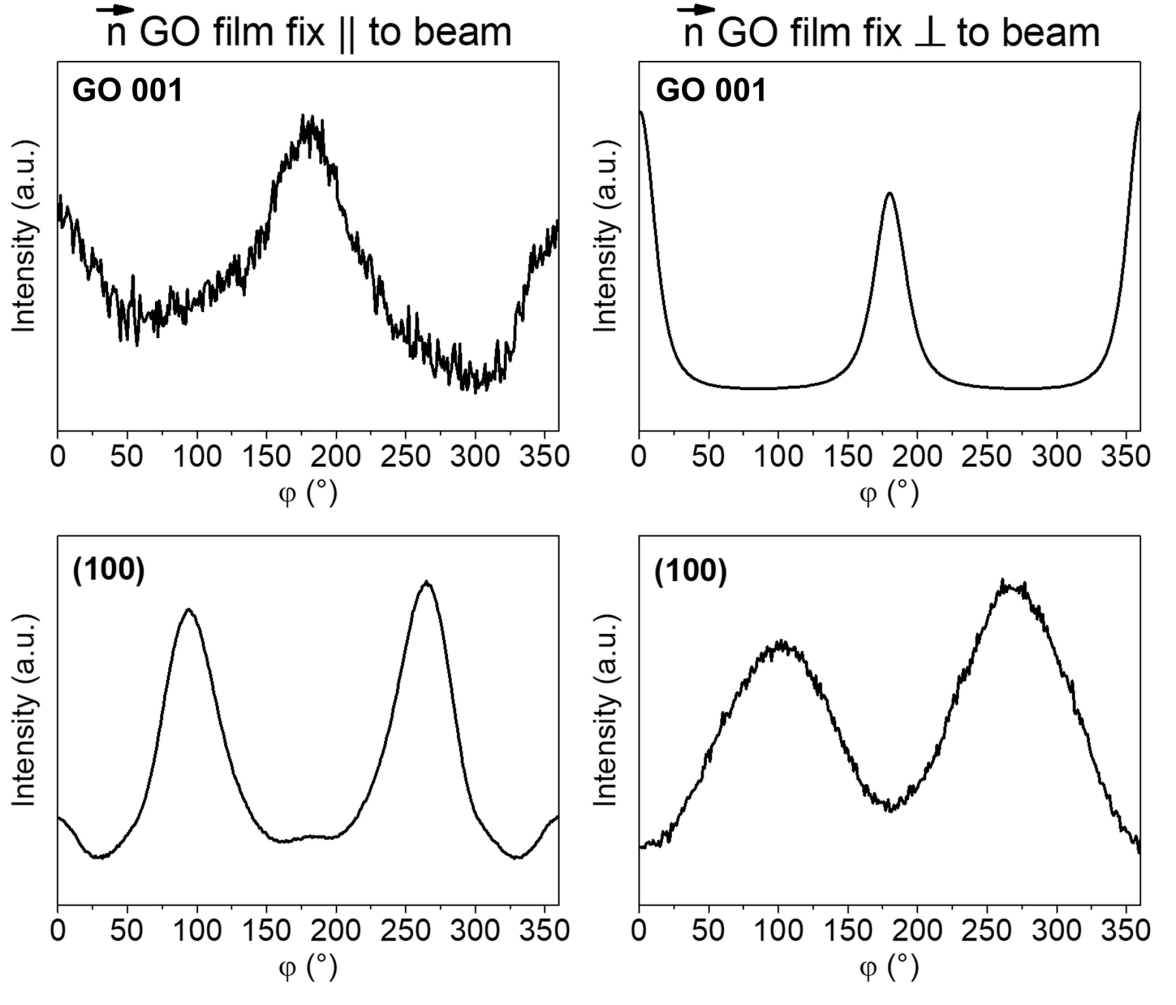
Comparison of the azimuthal integration profiles of the GO 001 and (100) scattering signals from the WAXS patterns shown in Fig. 5, corresponding to GO films oriented with the normal vector **n** either perpendicular or parallel to the incident X-ray beam.

First, we define the normal vector **n** to the GO film plane, which represents the average orientation of all individual GO flakes within the film. The GO film was then oriented such that this vector **n** was aligned either parallel or perpendicular to the incident X-ray beam [Fig. 5 ▸(*e*)]. To quantify the meso­structural anisotropy among the flakes, we employed WAXS measurements focused on GO 001 after the azimuthal integration (Fig. 6 ▸), which allowed for the calculation of the orientation distribution coefficient *S* for both sample orientations with respect to the beam.

Recent studies have highlighted the importance of comparative descriptors to evaluate anisotropy in GO assemblies. Li *et al.* (2020 ▸) introduced the anisotropic ratio from Herman’s order parameter quotient of *f*_∥_/*f*_⊥_ as a practical way of monitoring anisotropic enhancement during plasticization stretching of GO laminates, demonstrating that a relative parameter between orthogonal geometries provides clearer insights than absolute values alone. In contrast, Xin *et al.* (2019 ▸) quantified the Herman order parameter *S* for GO sheets assembled into fibers and belts, but their analysis was restricted to single orientations defined by the processing geometry, without a direct comparison between perpendicular and parallel directions within the same sample. Building on these approaches and following Li *et al.*’s dimensionless definition of the anisotropy ratio we define *R* as
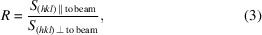
which condenses the relative alignment information into a single value. This normalization not only distinguishes isotropic (*R* = 1) from anisotropic arrangements (*R* = 0) but also reduces ambiguities in the interpretation of absolute orientation parameters, thereby complementing the conventional metrics of Herman’s order parameter.

Notably, the WAXS signal from the integrated diffractograms for GO films with **n** parallel and perpendicular to the X-ray beam [Figs. 5 ▸(*b*) and 5 ▸(*d*), respectively] showed a corresponding decrease and increase in the GO 001 peak intensity. This contrasts with the (100) peak intensity observed for the randomly oriented GO film. Since the GO 001 and (100) signals are orthogonal with respect to the internal structure of the flakes and the interface arrangement, their intensity ratio under isotropic conditions is expected to be approximately 3.3, as observed in the integrated pattern of the randomly oriented sample [Fig. 3 ▸(*b*)].

However, due to the drop-casting deposition method and the self-assembly process during film drying, the meso­structural ordering achieved deviates from randomness. For the oriented GO film, the calculated value of *R* for the GO 001 signal is summarized in Table 1 ▸.

The values of *R* obtained in this study are consistent with partial mesoscopic alignment and indicate a certain degree of anisotropy. Although drop casting is typically associated with random flake orientation, our results confirm that self-assembly effects during drying can induce significant structural anisotropy in the absence of external fields for sheet alignment. These findings highlight the sensitivity of WAXS-based orientation analysis and suggest its applicability for assessing flake alignment in other 2D systems, including MXenes or layered hybrid nanocomposites beyond GO. The methodology presented here thus offers a generalizable framework for quantifying interlayer alignment.

Although the values of *R* obtained do not approach the theoretical limit of perfect alignment (*R* = 0), they clearly deviate from the isotropic case (*R* = 1), confirming the presence of mesoscopic ordering. Given the absence of external aligning forces during film formation, these results indicate that intrinsic factors, such as van der Waals inter­actions, capillary flow during solvent evaporation and interflake electrostatic repulsion, might contribute significantly to the preferential orientation of GO flakes. Further insights into these mechanisms could be obtained by combining WAXS orientation parameters with complementary analyses, such as polarized optical microscopy birefringence measurements or molecular dynamics simulations. Such multimodal approaches may support the establishment of WAXS-based metrics as a quantitative standard for evaluating orientational ordering in GO and other lyotropic two-dimensional systems.

## 5. Conclusions

This study demonstrates that WAXS can be used to give a quantitative assessment of the degree of orientational ordering among flakes in GO films and not only to extract conventional orientation parameters such as *S* or *f*_H_. By measuring the WAXS signal from two sample orientations parallel and perpendicular to the incident X-ray beam, it was possible to calculate the orientation distribution coefficient *S* for the GO 001 and (100) reflections. From these, the anisotropy ratio *R* was derived to characterize the mesoscopic alignment of the GO flakes in the GO 001 signal. This resolves ambiguities inherent in single-geometry measurements and provides a more intuitive indicator to distinguish isotropic from anisotropic flake arrangements. This approach reinforces the value of WAXS as a versatile tool for the structural analysis of partially ordered layered systems.

The results indicate that, even in films prepared by simple drop casting, which are typically expected to exhibit random orientation, the self-assembly process during solvent evaporation induces partial anisotropy. This was shown by the non-unity values of *R*, which deviate significantly from the isotropic case.

These findings contribute to the fundamental understanding of mesostructural ordering in 2D materials and suggest that even passive deposition methods, such as drop casting, can lead to spontaneous alignment under certain drying conditions. Future work may investigate the influence of substrate surface properties, concentration gradients and interfacial shear on the emergence and control of anisotropic domains within flake-based films.

Furthermore, the GO 001/(100) intensity ratio under isotropic and anisotropic conditions may serve as a structural fingerprint for assessing orientational order in flake-based nanomaterials.

## Supplementary Material

GO sample preparation in the WAXS sample holder. DOI: 10.1107/S1600576725008714/uu5016sup1.pdf

Azimuth integration video record employing FIT2D software. DOI: 10.1107/S1600576725008714/uu5016sup2.mp4

Example of the output data generated with FIT2D, G1001 azimuth. DOI: 10.1107/S1600576725008714/uu5016sup3.txt

## Figures and Tables

**Table 1:** Orientation distribution coefficient *S* and Herman’s orientation function *f*_H_ for the different WAXS scattering signals of GO films oriented either parallel or perpendicular to the incident X-ray beam

	WAXS feature	*S*	〈cos^2^ φ〉	*R* [equation (3)]
**n** GO film ∥ to beam	GO 001	0.05	0.37	0.11
	(100)	−0.13	0.24	
**n** GO film ⊥ to beam	GO 001	0.46	0.64	
	(100)	−0.02	0.31	

## Data Availability

The data supporting the findings of this study are available from the corresponding author upon reasonable request.

## Footnotes

1 In this work, we use ‘GO 001’ without brackets to denote the interflake spacing signal of graphene oxide, which has no true crystallographic indexing. Parentheses (*hkl*) are used for reflections originating from actual crystallographic planes (*e.g.* in graphite).
