# Supplementary material for: Quantifying interflake ordering in graphene oxide films via wide-angle X-ray scattering analysis
Source: J Appl Crystallogr. 2025 Nov 26;58(Pt 6):2105–11. doi: 10.1107/S1600576725008714 (PMC12810526; doi:10.1107/S1600576725008714)
Supplement: Supplementary file 1 [file j-58-02105-sup1.pdf]

JOURNAL OF
APPLIED
CRYSTALLOGRAPHY

Volume 58 (2025)

Supporting information for article:

Quantifying interflake ordering in graphene oxide films via wide-angle X-ray scattering analysis

Roque Sanchez Salas, Aaron Morelos Gomez, David Angel Sanchez Hernandez and Sandra Loera Serna

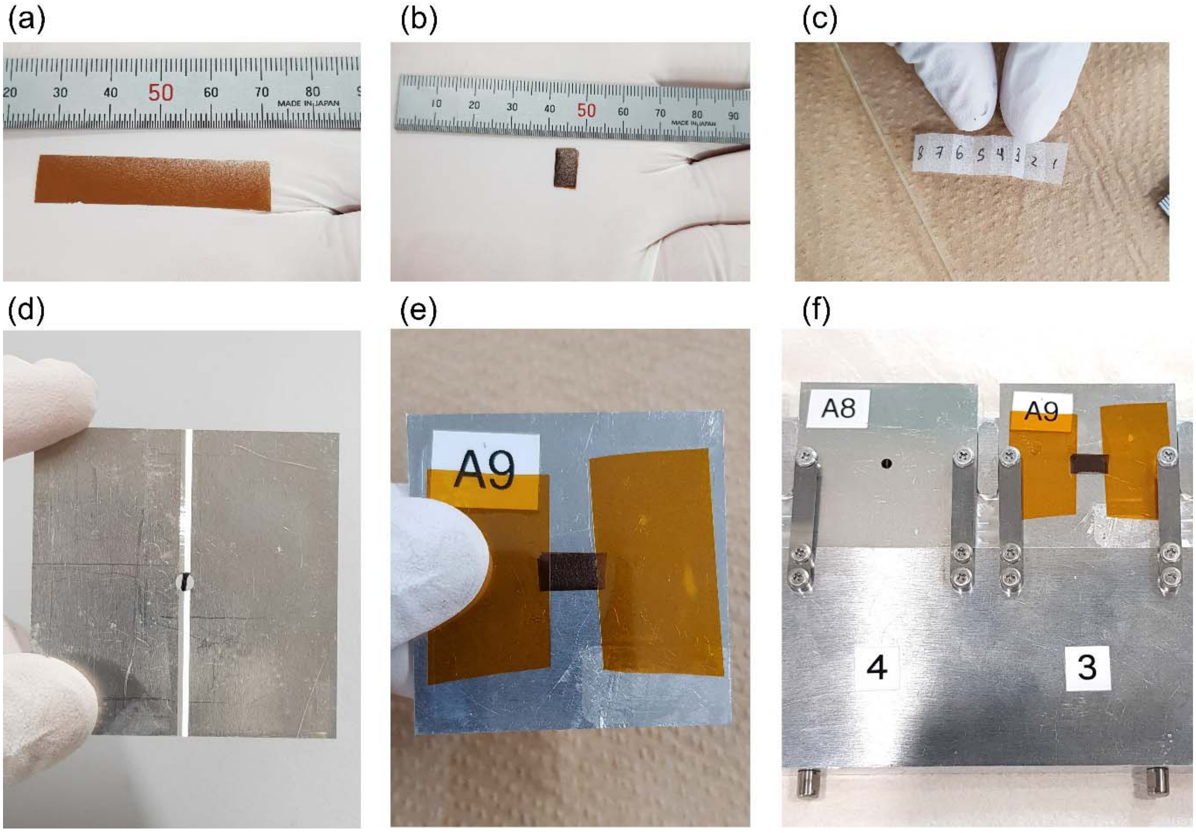

Figure S1 (a) GO film sample dimensions approx. 4x1 cm; (b) folded GO film into 8 layers with similar fold dimensions (approx. 5mmx10mm) as shown in (c) for better visualization; (d) a piece of folded GO film was hand manipulated with tweezers and fixed by pressure without any Kapton tape into the hole of WAXS sample holder with a diameter of 3 mm, this set of GO film corresponds to a perpendicular direction of the normal vector of the film respect to the beam; (e) folded GO film was fixed from both sides but without any Kapton tape in the centre hole diameter (3 mm) of the WAXS sample holder, this set of GO film corresponds to a parallel direction of normal vector of the film respect to the beam; (f) GO film samples and WAXS sample holder in position to be measured, left and right hand correspond to perpendicular and parallel direction of the normal vector of the film respect to the beam, respectively.
